# Clival Chondrosarcoma Associated With an Intra-Axial Cystic Medullary Lesion Responsive to Steroids

**DOI:** 10.3389/fneur.2018.00502

**Published:** 2018-06-26

**Authors:** Johannes Walter, Sandra Kapitza, Niklaus Krayenbühl, Alexander A. Tarnutzer

**Affiliations:** ^1^Department of Neurosurgery, University Hospital Zurich, Zurich, Switzerland; ^2^Faculty of Medicine, University of Zurich, Zurich, Switzerland

**Keywords:** medulla oblongata, inflammation, brainstem, surgery, clivus, chondrosarcoma, dexamethasone

## Abstract

**Introduction:** Here we present a 75-year-old patient who was admitted with acute-onset right-sided hemiparesis, dysphagia, dysarthria and nystagmus. Repeated MRI scans showed two lesions with contact to one another: one solid stationary extra-axial lesion at the caudal part of the clivus and a rapidly growing intra-axial cystic lesion at the level of the medulla oblongata. Biopsy of the solid lesion demonstrated a low-grade chondrosarcoma, while no tissue sample of the cystic lesion could be retrieved. After initiation of dexamethasone therapy the cystic lesion markedly regressed.

**Background:** A literature search on published cases with the same combination of a stationary solid extra-axial mass at the caudal part of the clivus and a growing intra-axial cystic mass in the medulla oblongata was negative, indicating that the case described here is both unique and novel.

**Discussion:** Considering the rapid progression of symptoms and growth on MR-imaging in combination with the marked response to steroids, an inflammatory response linked to the chondrosarcoma is most likely. At the same time other possible explanations as a second neoplasm, an abscess or an ischemic lesion seem unlikely.

**Concluding remarks:** This case underlines an unusual complication of a rare brainstem tumor and outlines both the differential diagnosis and potential treatment options. For such cystic lesions in combination with chondrosarcoma, a treatment course with steroids should be considered along with surgical exploration necessary to obtain the diagnosis and for potential reduction of mass-effect on the medulla oblongata.

## Introduction

**Figure 1:**
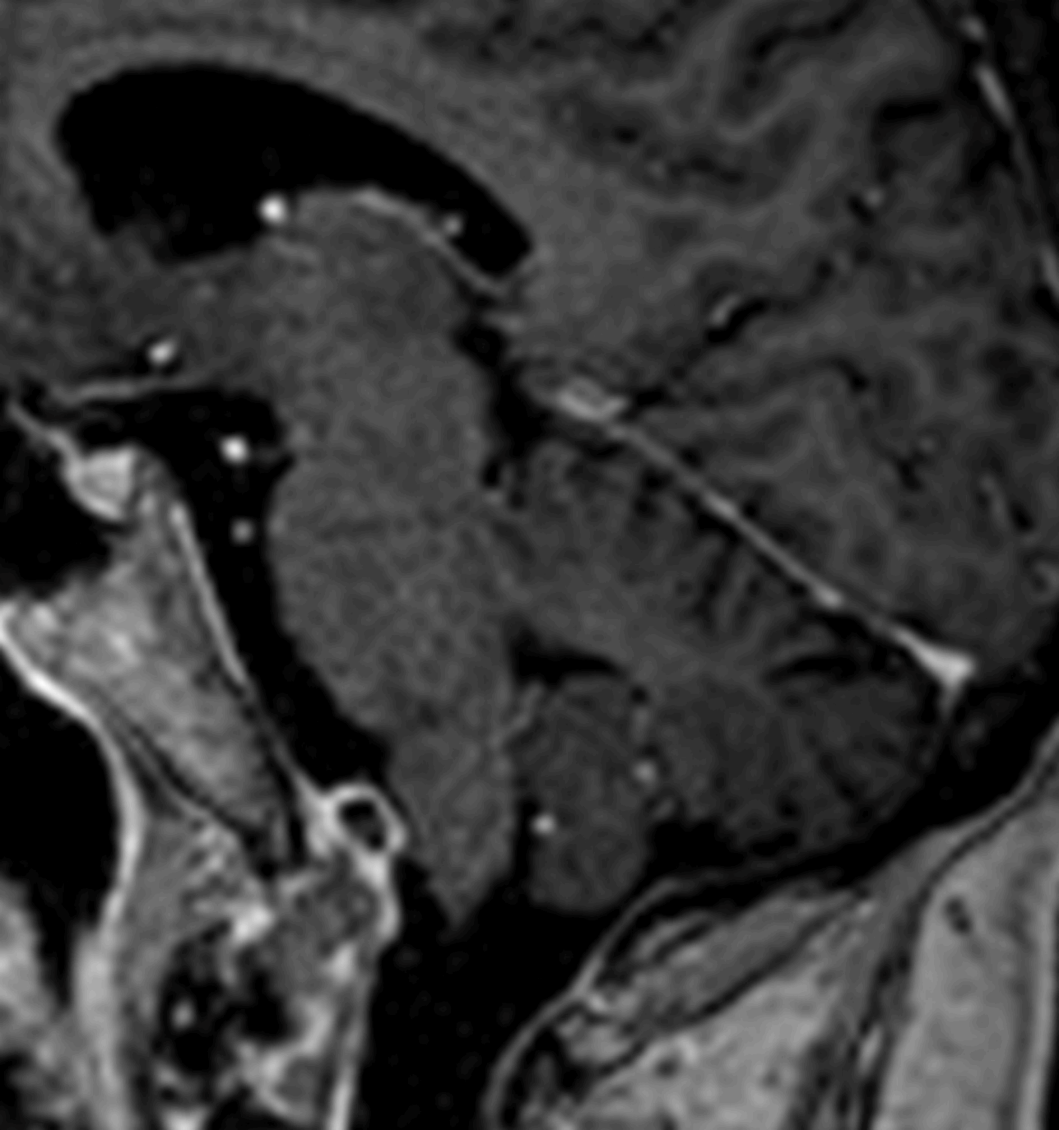
Sagittal, contrast-enhanced T1-weighted MR-image showing a mass (7 × 8 × 15 mm, height x depth x width) with peripheral contrast-uptake that was later identified as a low-grade chondrosarcoma. This mass was attached to the clivus and was located in close proximity to the medulla oblongata. Note that this MR-image was taken on day 2 and remained stationary over the course of the next 2 months.

**Figure 2:**
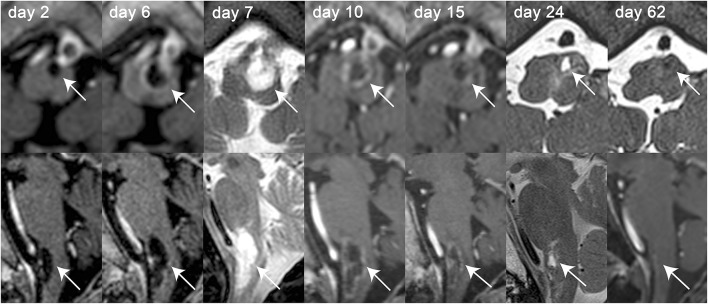
Axial (top row) and sagittal (bottom row) MR-images of the intra-axial cystic lesion (indicated by the white solid arrows) located in the left part of the medulla oblongata over the course of 2 months. MR-images were obtained on day 2, 6, 7, 10, 15, 24, and 62 after symptom onset as indicated on the single MR-images. Whenever available, T1-weighted contrast-enhanced MR-images were selected for both axial and sagittal images [day 2, 6, 10, 15, 62 (only sagittal image)]. For the other MR-sessions, either T2-weighted (day 7) or gradient-echo T1-weighted [day 24 and 62 (only axial image)] MR-images were selected. Over the course of disease growth [from 16 × 5 × 4 mm on day 2–25 × 11 × 9mm on day 10 (height × depth × width)] and contrast-enhancement (first noted on day 6) of the cystic lesion can be depicted. After treatment initiation with dexamethasone (day 9) and surgical preparation of the solid lesion (day 15), the size of the cystic lesion decreased continuously and could not be detected any more on day 62.

A 75-year-old womanwas admitted to another hospital with progressive right-sided hemiparesis for 24 h. She had no headache, nausea, vomiting, or dizziness. On examination, slight facial asymmetry and mild-to-moderate right-sided hemiparesis were noted. A non-contrast CT-scan (as she was allergic to iodine) showed no signs of intracranial hemorrhage, ischemia or mass lesions. On the next day, an MRI of the brain was obtained. Besides moderate-to-severe chronic vascular leukoencephalopathy a cystic, T2-hyperintense intra-axial lesion (Figure 1) in the medulla oblongata (16 × 5 × 4 mm, height × depth × width) and a contrast-enhancing extra-axial lesion at the caudal part of the clivus (7 × 8 × 15 mm) were noted (Figure 2). There were no signs of acute ischemia [diffusion-weighed imaging (DWI) negative, no decrease in apparent diffusion coefficient (ADC)] or bleeding (susceptibility weighted imaging (SWI) negative). On day 3 the right-sided hemiparesis worsened and dysarthria, dysphagia, tongue-deviation to the left and skew deviation with diplopia developed. The MRI-scan was repeated (day 6), demonstrating growth of the cystic lesion (27 × 9 × 7 mm) with contrast-enhancement of its posterior wall. The clival lesion was unchanged and had a close spatial relationship to the other lesion. Another MRI (day 7) showed further progression of the cystic lesion (27 × 9 × 8 mm). On day 9 the patient was transferred to this hospital.

**Figure 3:**
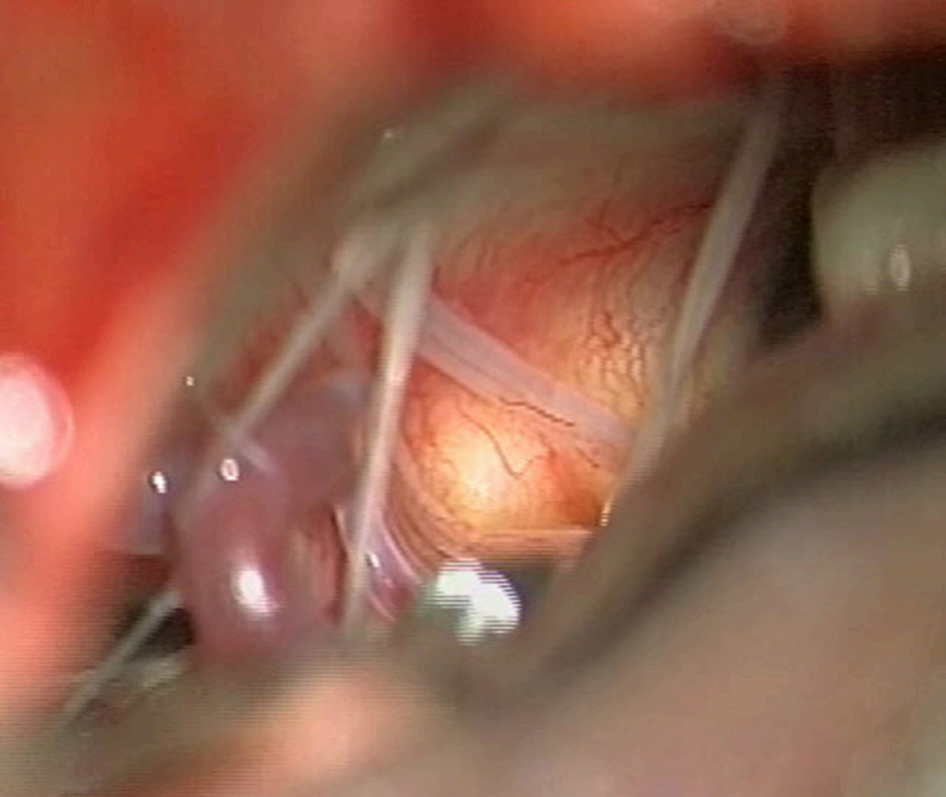
Intraoperative view on the left inferior cerebellopontine angle (day 15). The bulging of the vascularized dura over the tumor can be seen and the lower cranial nerves are displaced. The posterior inferior cerebellar artery (PICA) is seen on the inferior aspect.

On physical examination, the patient had slightly anisocoric pupils (right>left) equally reactive to light, saccadic smooth pursuit and hypometric saccades, gaze-evoked nystagmus, upbeat-nystagmus, right-sided ptosis, right-sided weakness of the buccal branch of the facial nerve, dysarthria, dysphagia, uvula, and tongue deviation to the left and moderate-to-severe right-sided hemiparesis with a positive Babinski sign. A lumbar puncture showed 6 cells/μl (predominantly mononuclear, normal range = 0–4 cells/μl) and slightly elevated protein levels (0.508 g/l, normal range = 0.2–0.4 g/l). The increased cell count and the elevated protein level were suggestive of an inflammatory response and therefore dexamethasone was started. Repeated MRI (day 10) showed peripheral contrast-enhancement of the cystic lesion (25 × 11 × 9 mm) and progressive perifocal edema (Figure 1), whereas 5 days later (day 15) slight regression of the cystic lesion (22 × 6 × 6 mm) was noted. On the same day after MR-imaging (day 15) a lateral sub-occipital craniotomy was performed. Intraoperatively, the two lesions seemed to be connected and the clival lesion was tightly adherent to the dura mater (Figure 3), which made it very difficult to explore the lesion in the medulla oblongata. Specimens were taken only from the extra-axial lesion and a diagnosis of a low-grade chondrosarcoma was made. Postoperatively, the right-sided hemiparesis transiently worsened but improved again within the next 2 weeks. An MRI performed 9 days postoperatively (day 25) showed regression of the cystic mass (6 × 4 × 3 mm). The chondrosarcoma was unchanged. The patient was discharged to a rehabilitation facility on the fifteenth postoperative day (day 31) and dexamethasone was stopped. On follow-up assessment 5 weeks later, the patient was able to walk with a wheeled walker, dysphagia had markedly improved, but dysarthria persisted. On MRI (day 62) the chondrosarcoma was unchanged and the cystic lesion had almost completely disappeared. Lumbar puncture was repeated, showing 1 cell/μl and a protein content of 0.780 g/l (normal range = 0.2–0.5 g/l). Cytopathologic analysis demonstrated no malignant cells. There was evidence of oligoclonal bands both in the cerebrospinal fluid and the blood serum, suggesting status post a systemic immunoreaction. After 3 days, the patient returned to the rehabilitation facility.

The case was discussed at the multidisciplinary tumor board and the patient was advised to undergo radiation therapy for the remaining chondrosarcoma. Fractioned proton radiation therapy (total dose 70Gy, 35 sessions of 2Gy each) was completed 6 months after the diagnosis was made and since then no further disease progression was noted.

No ethical approval was obtained as this was a single case report. Written informed consent for publication of this case report was retrieved from the patient.

## Background

We performed a literature search on published cases with the same combination of a stationary solid extra-axial mass at the caudal part of the clivus and a growing intra-axial cystic mass in the medulla oblongata. However, to our best knowledge, we could not identify comparable cases previously described in the literature. As the differential diagnosis of the case presented here is the main focus of this manuscript, we will address previously published cases with certain similarities in the Discussion section.

## Discussion

This 75-year-old patient presented with rapidly progressive hemiparesis, nystagmus, dysphagia, and dysarthria due to a growing intra-axial cystic mass in the medulla oblongata as well as a stationary solid extra-axial mass at the caudal part of the clivus identified as chondrosarcoma. Based on a recently published case series of isolated medullary lesions, we categorized the intra-axial lesion as suggested by Prakkamakul et al. (1). Presenting as a single cystic lesion, a neoplasm seems most likely. However, in their case series no combined solid extra-axial and cystic intra-axial medullary lesion was described and also no case with low-grade chondrosarcoma was reported. Therefore, the intra-axial cystic lesion may not reflect a neoplasm itself but could be an inflammatory response secondary to an extra-axial tumor. This, however, is not reflected in the proposed classification by Prakkamakul et al. (1). Thus, as no histologic diagnosis of the cystic lesion could be obtained, still several possible explanations have to be considered.

This includes a second, independent intra-axial neoplasm. Since the cystic lesion responded to dexamethasone and with absent clinical and laboratory features of systemic lymphoma, the first suspicion was lymphoma of the central nervous system (PCNSL). PCNSL are a rare form of lymphoma and very rarely located in the brainstem. They usually show homogenous contrast-enhancement, but ring-like contrast-enhancement is possible (2). This diagnosis was unlikely, however, because the cystic lesion did not re-emerge after stopping dexamethasone and it was negative on diffusion-weighted imaging. Likewise, astrocytomas of the medulla oblongata can show ring-like contrast-enhancement and cystic portions. However, brainstem astrocytomas are rare and all reported cases had a nodulous portion on MRI (3), which was not the case here. Furthermore, regression of the cystic mass under dexamethasone basically rules out astrocytoma.

Due to the cystic appearance, an endodermal cyst, abscess or hemangioblastoma should be included in the differential diagnosis. Endodermal cysts are usually located extra-axially in the posterior fossa (4), whereas a few cases of intra-axial endodermal cysts in the medulla oblongata have been reported (5). The median age of patients with intra-axial endodermal cysts was 27 years and there is no reported complete regression of an endodermal cyst under steroids. Hemangioblastoma, which can appear cystic on MRI, are rarely located in the medulla oblongata and always have a mural nodule, which was not present in this case (6). Due to the ring-like contrast enhancement on MRI, an intra-axial abscess was considered. However, diffusion-weighted imaging was negative and there were no signs of inflammation in the cerebrospinal fluid. Furthermore, the lesion completely regressed on dexamethasone, which rules out the diagnosis of an abscess.

The rapid onset would be consistent with an underlying vascular pathology such as brainstem ischemia or hemorrhage (possibly secondary to a vascular malformation). Yet, the progressive clinical course and MR-imaging do not support this etiology. With the intra-axial lesion being hyperintense on T2 rather than hypointense (as expected for vascular malformation) and being negative on SWI-weighted imaging, this speaks against a vascular pathology. Furthermore, such a vascular lesion would not have resolved by administration of dexamethasone or surgical decompression of the medulla (7, 8).

Alternatively, the cystic lesion could have been caused by an inflammatory process as the cerebrospinal fluid cell count was slightly elevated and as the patient improved clinically and radiologically on dexamethasone. The inflammatory process could have been caused by compression of the medulla oblongata due to the mass effect of the solid clival lesion. This hypothesis is supported by the fact, that the cystic lesion regressed after surgical reduction of the mass effect on the medulla oblongata. However, regression already started before the surgical intervention. Therefore, rather than pressure, induction of inflammatory processes by the chondrosarcoma itself must be considered as well. Even though unusual, tumor-associated infiltration of inflammatory cells has been linked to chondrosarcomas (9, 10). Furthermore, paraneoplastic inflammatory phenomena such as antibody-mediated encephalitis and vasculitis have been described in chondrosarcoma (11, 12). Even though there has been no reported case of a chondrosarcoma-induced cystic lesion in the literature, an induction of inflammation by both mass effect and infiltration of inflammatory cells seems the most likely etiology.

## Concluding remarks

In conclusion, in this 75-year-old patient with a cystic intramedullary mass and a clival chondrosarcoma causing symptoms of the lower cranial nerves and hemiparesis, the most plausible etiology of the cystic lesion is an inflammatory process as for several reasons two distinct neoplasms or underlying vascular pathologies seem highly unlikely. For such cystic lesions in combination with chondrosarcoma, a treatment course with steroids should be considered along with surgical exploration necessary to obtain the diagnosis and for potential reduction of mass-effect on the medulla oblongata.

## Author contributions

JW was involved in the conceptualization of the case report, the clinical assessment of the patient, drafted and critically reviewed the manuscript. SK was involved in the clinical assessment of the patient and critically reviewed and edited the manuscript for content. NK performed the surgery, contributed the intraoperative images and critically reviewed and edited the manuscript for content. AT was involved in the conceptualization of the case report, the clinical assessment of the patient and critically reviewed and edited the manuscript for content. All authors read and approved the final version of the manuscript.

### Conflict of interest statement

The authors declare that the research was conducted in the absence of any commercial or financial relationships that could be construed as a potential conflict of interest.
